# Interaction of silver nanoparticles with algae and fish cells: a side by side comparison

**DOI:** 10.1186/s12951-017-0254-9

**Published:** 2017-02-28

**Authors:** Yang Yue, Xiaomei Li, Laura Sigg, Marc J-F Suter, Smitha Pillai, Renata Behra, Kristin Schirmer

**Affiliations:** 1 Department of Environmental Toxicology, Eawag, Swiss Federal Institute of Aquatic Science and Technology, 8600 Dübendorf, Switzerland; 2 School of Architecture, Civil and Environmental Engineering, École Polytechnique Fédérale de Lausanne, 1015 Lausanne, Switzerland; 3 Department of Environmental Systems Science (D-USYS), ETH-Zürich, 8092 Zürich, Switzerland; 4 Department of Basic Sciences and Aquatic Medicine, Norwegian University of Life Sciences (NMBU), Oslo, 0454 Norway; 5 Wattstrasse 13a, 8307 Effretikon, Switzerland

**Keywords:** AgNP, *Euglena gracilis*, RTgill-W1 cell line, Nanoparticle uptake, Nanoparticle toxicity, Nanoparticle-protein interactions

## Abstract

**Background:**

Silver nanoparticles (AgNP) are widely applied and can, upon use, be released into the aquatic environment. This raises concerns about potential impacts of AgNP on aquatic organisms. We here present a side by side comparison of the interaction of AgNP with two contrasting cell types: algal cells, using the algae *Euglena gracilis* as model, and fish cells, a cell line originating from rainbow trout (*Oncorhynchus mykiss*) gill (RTgill-W1). The comparison is based on the AgNP behavior in exposure media, toxicity, uptake and interaction with proteins.

**Results:**

(1) The composition of exposure media affected AgNP behavior and toxicity to algae and fish cells. (2) The toxicity of AgNP to algae was mediated by dissolved silver while nanoparticle specific effects in addition to dissolved silver contributed to the toxicity of AgNP to fish cells. (3) AgNP did not enter into algal cells; they only adsorbed onto the cell surface. In contrast, AgNP were taken up by fish cells via endocytic pathways. (4) AgNP can bind to both extracellular and intracellular proteins and inhibit enzyme activity.

**Conclusion:**

Our results showed that fish cells take up AgNP in contrast to algal cells, where AgNP sorbed onto the cell surface, which indicates that the cell wall of algae is a barrier to particle uptake. This particle behaviour results in different responses to AgNP exposure in algae and fish cells. Yet, proteins from both cell types can be affected by AgNP exposure: for algae, extracellular proteins secreted from cells for, e.g., nutrient acquisition. For fish cells, intracellular and/or membrane-bound proteins, such as the Na^+^/K^+^-ATPase, are susceptible to AgNP binding and functional impairment.

**Electronic supplementary material:**

The online version of this article (doi:10.1186/s12951-017-0254-9) contains supplementary material, which is available to authorized users.

## Background

Owing to their unique antimicrobial properties, silver nanoparticles (AgNP) are among the most widely used engineered nanoparticles in a variety of consumer products and medical applications, such as textiles and paints. With washing, rain and through other routes, these nanoparticles can be released into the environment, especially into the aquatic environment [1]. This raises concern about potential adverse effects in aquatic organisms. On this background, the toxicity of AgNP to aquatic organisms has been tested on a variety of organisms, ranging from bacteria, to plants, fungi, algae, invertebrates and fish [2–4]. However, with few exceptions [5, 6], most studies did not clearly attribute toxicity to either direct effects of AgNP or to indirect effects of dissolved silver, which includes all the silver species in oxidized state Ag(I) in aqueous solution, such as Ag^+^, AgCl_n_ (aq) and AgOH (aq), stemming from AgNP.

Among aquatic organisms, algae and fish are two important models. As autotrophic organisms, algae are primary producers, i.e. they fix CO_2_ to produce oxygen in the presence of light. They are at the base of the food chain, serving as food to, e.g. water flea but also fish. Microalgae are single cell organisms surrounded by an inner plasma membrane and an outer semi-permeable cell wall of various compositions. The pores in such cell walls have a size estimated to be 5–20 nm. It helps the algae to maintain integrity and constitutes a primary site for interaction with the surrounding environment [7]. Algae connect with their environment by releasing, e.g. digestive enzymes, for nutrient acquisition. Whether algae have sophisticated mechanisms of particle uptake, such as via endocytosis (see below), is still a matter of debate. Accordingly, internalization of nanoparticles in algae was suggested in only a few studies [8, 9]. There was no evidence of nanoparticle uptake into algae in many other studies using electron microscope imaging and/or analysis of internalized metal in cells [10–14]. These findings emphasize the role of the algal surface as a potential barrier against nanoparticle entry into the cells, with the limitation likely being the pore size in the cell wall.

In contrast to microalgae, fish are heterotrophic, multiple organ- and tissue-based organisms. Fish are at a higher trophic level than algae but depend on the oxygen that algae and other autotrophic organisms produce. Depending on the species, fish can be consumers of algae or of other heterotrophs. With respect to environmental exposure to chemicals or nanoparticles, the fish gill is an important interface due to its large surface. The gill affords gas exchange between the external water environment and internal environment of the organism. In this exchange process, other substances, like metal nanoparticles and organic compounds, can interact with fish gill cells and eventually pass into the blood stream. Therefore, the fish gill can be considered a target of fish-nanoparticle interactions. Accordingly, AgNP were found to be most highly concentrated within gill and liver tissue of rainbow trout (*Oncorhynchus mykiss*) after a 10-day exposure [15]. In contrast to algae cells, fish gill cells, like all animal cells, are cell wall-free. Several kinds of endocytic pathways were proposed for nanoparticle incorporation into animal cells: clathrin-mediated endocytosis, caveolae-mediated endocytosis, macropinocytosis and phagocytosis [14, 16]. Once the vesicles carrying nanoparticles are internalized and detach from the plasma membrane, the vesicles are sorted and transported to different endocytic compartments. By these processes, nanoparticles are delivered to other subcellular compartments in endocytic pathways, from early endosome and multi-vesicular bodies to late endosomes and lysosomes [17].

Independent of the mechanism of particle uptake, nanoparticles tend to bind molecules from the surrounding environment owing to their big surface-to-mass ratio. During nanoparticle interaction with cells, proteins are an important class of biomolecules that are prone to binding to nanoparticles, leading to a protein corona [18, 19]. With regard to extracellular proteins, such as the digestive proteins excreted by algae and bacteria, a so-called “eco-corona” can form [20, 21]. Intracellular proteins, on the other hand, can bind to particles upon uptake into cells. With the binding to nanoparticles, the properties and functions of proteins can change compared to unbound proteins. Thus, it is also important to understand to what extent nanoparticle-protein complexes impact on the properties of the proteins. Studies on the nanoparticle-protein interactions initially focused on single proteins. For example, Wigginton [22] found that AgNP inhibited tryptophanase (TNase) activity in the interaction with *E. coli* proteins and a dose-dependent inhibition of enzyme activity was observed for the incubation of citrate-coated AgNP with firefly luciferase [23]. In contrast to single protein-nanoparticle interactions, only few studies have thus far focused on identifying proteins that bind out of a complex mixture, especially in an intact intracellular environment [24, 25]. Such studies not only help identify the proteins most susceptible to particle binding but can also guide future research on single protein-particle interactions.

In order to shed light on the detailed mechanisms of interaction between AgNP and cells of algae and fish, we explored different aspects of AgNP-cell interactions, spanning AgNP behavior in exposure media, toxicity to cells, uptake and interaction with proteins. We aimed to critically compare the interaction of AgNP with contrasting cell types belonging to autotrophic vs. heterotrophic organisms in order to support a rational assessment of risks based on our previous studies [26–29]. A species of algae, *Euglena gracilis*, and a fish gill cell line, RTgill-W1 [30], originating from rainbow trout (*Oncorhynchus mykiss*), were selected to represent an autotrophic and a heterotrophic aquatic cellular system. The *Euglena gracilis* has no rigid cell wall but a flexible glycoprotein-containing pellicle, which aligns on the surface in longitudinal articulated stripes [31]. It was selected on purpose because nanoparticle uptake was thought to more likely occur in such an algae compared to one with a rigid cell wall. The RTgill-W1 cell line can survive in a simplified exposure medium, which provides the possibility to expose cells in medium that more closely mimics the aqueous environment a fish gill would face [32, 33]. Both algae and fish gill cell exposures were performed in minimal media supporting cell survival but not proliferation, in order to provide better controllable exposure and effect assessment for mechanistic studies. Here we focus on the comparative aspects of the outcome of our research. Unless noted otherwise, we will refer to *E. gracilis* as “algal cells” and to the RTgill-W1 fish gill cell line as “fish cells”.

## Results and discussion

### The composition of exposure media significantly influences AgNP behavior

The size, zeta potential and dissolution of AgNP were tested over time in exposure media for algae and fish cells (Table 1). To avoid silver complexation, only 10 mM 3-morpholinopropanesulfonic acid (MOPS, pH 7.5) was used as exposure medium in algae experiments [26]. In the stock solution, the initial Z-average size and zeta potential of AgNP were 19.4 nm and −30 mV, respectively. AgNP were stable in this medium with an average size of 38–73 nm and a zeta potential of −23 to −28 mV up to 4 h of incubation [26]. For the fish cells, three kinds of exposure media were selected: L-15/ex, a regular, high ionic strength and high chloride cell culture medium based on Leibovitz’ 15 (L-15) [32, 34]; L-15/ex w/o Cl, a medium without chloride to avoid the formation of AgCl and study the role of chloride in silver ion and AgNP toxicity; and d-L-15/ex, a low ionic strength medium that more closely mimics freshwater [27]. The AgNP moderately agglomerated (average size: 200–500 nm; Zeta potential: −15 mV) in L-15/ex medium. In L-15/ex w/o Cl medium, AgNP strongly agglomerated with an average size of 1000–1750 nm and a zeta potential of −10 mV. In d-L-15/ex medium, AgNP dispersed very well (average size: 40–100 nm; Zeta potential: −20 mV). Even though the size of AgNP increased up to 1750 nm, we found that large size AgNP were due to agglomeration [27], which is a reversible process and AgNP can easily be dispersed again [35]. The UV–Vis absorbance of AgNP in exposure media confirmed the different behavior of AgNP in the different media [26, 27]. Transmission electron microscopy (TEM) images of fish cells showed that single or slightly agglomerated AgNP were located in endosomes and lysosomes in fish cells, which indicates that fish cells took up AgNP in nanoscale [28].

**Table 1:** AgNP behavior in exposure media for algae and fish cells

	Algae exposure medium [26]	Fish cell exposure media [27]		
		L-15/ex	L-15/ex w/o Cl	d-L-15/ex
Medium ionic strength (mM)	3.44	173.0	177.1	72.0
Size of AgNP (nm)	38–73	200–500	1000–1750	40–100
Zeta potential of AgNP (mV)	−23 to −28	−15	−10	−20
Dissolution of AgNP (% of total Ag)^a^	1.7%	1.89%	0.67%	0.40%

^a^The level of dissolution of AgNP represents the mean of dissolution data obtained using two different methods to separate dissolved silver from particles: ultrafiltration and ultracentrifugation. Values given are the mean of the average data obtained for each method, carried out three independent times

The dissolution of AgNP, expressed as percentage of free to total silver, was comparable in MOPS and L-15/ex (~1.8%); dissolution was somewhat lower in L-15/ex w/o Cl and d-L-15/ex medium (~0.5%). Depending on the applied concentrations, this amounts to dissolved silver in the range of 1 nM to 2 µM (assuming 1–2% dissolution in 0.1–100 µM AgNP suspension). Upon contact with algae or fish cells, the uptake of dissolved silver shifts the AgNP/silver ion equilibrium and more silver ions are released. Furthermore, previous work reported that AgNP accumulated in mammalian cell endosomes and lysosomes displayed higher dissolution in these acidic environments than in a neutral environment [17, 36]. Therefore, we expect significant dissolution of AgNP in this process and used AgNO_3_ as a dissolved silver control throughout.

The diverse behavior of AgNP in the different exposure media demonstrates the importance of accounting for nanoparticle characteristics in the respective exposure environments. The composition of the exposure media showed a strong influence, especially in terms of particle agglomeration but also in terms of dissolution. In high ionic strength medium, high concentrations of ions can break the electrical double layers surrounding the AgNP and thereby decrease the surface charge, which leads to AgNP agglomeration. In the presence of chloride, AgNP were more stable (compare L-15/ex medium to L-15/ex w/o Cl), which means chloride ions can stabilize AgNP, likely by binding to AgNP surfaces and contributing to a negative surface charge. In terms of AgNP dissolution, a higher percentage was found in L-15/ex with high chloride: chloride shifts the equilibrium of AgNP dissolution by complexing the dissolved silver.

### AgNP adsorb to the algal cell surface but can be taken up by fish cells

To quantitatively relate AgNP/AgNO_3_ exposure to the toxicity seen in algal and fish cells, cell-associated silver was quantified by inductively coupled plasma mass spectrometry (ICP-MS). Upon exposure to similar concentrations of AgNP or AgNO_3_, the cell-associated silver in algae cells was comparable with the cell-associated silver which was reported for the alga *Chlamydomonas reinhardtii* [11]. Similarly, the cell-associated silver in RTgill-W1 cells was also comparable with the silver content in other vertebrate cell types, such as mouse erythroleukemia cells [37] and HepG2 cells [38].

**Fig. 1:**
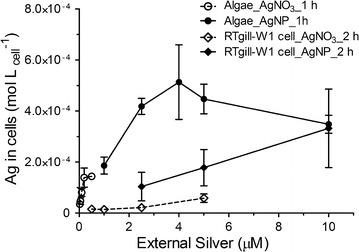
Cell-associated silver in algae and fish cells. Cell-associated silver levels (mol/L_cell_) were quantified by ICP-MS after exposure to AgNP and AgNO_3_ for 1 h (algae) and 2 h (fish cells). The exposure of the algal cells was in MOPS; that of the fish cells in d-L-15/ex medium. The concentrations of silver (AgNP, AgNO_3_) were selected based on the concentration response curves obtained for algae [26] and fish cells [28]. Cells were washed with cysteine solution to remove any loosely bound silver prior to extraction and analysis. Data presented as mean ± SD; n = 3

At comparable external AgNO_3_ exposure concentrations (0.1–0.5 µM), the silver content associated with algal cells was 2.4–4.2 times higher than in the fish cells (Fig. 1). This was probably due to the different compositions of the exposure media and the resulting different dissolved silver species. In the algal exposure medium, MOPS, almost all dissolved silver was present as free silver ions (Ag^+^) as predicted by Visual MINTEQ (V3.1, KTH, Sweden). Free silver ions are taken up via copper transporters in algae, as suggested in *C. reinhardtii*, *Pseudokirchneriella subcapitata* and *Chlorella pyrenoidosa* [39–41]. On the contrary, in fish cell exposure medium, only around 60% of dissolved silver was in the form of Ag^+^. The other 40% reacted with chloride and formed neutral or negatively charged complexes (AgCl_n_^(n−1)−^) [27]. Earlier research showed that Ag^+^ has a higher bioavailability than AgCl_n_^(n−1)−^ complexes in rainbow trout and Atlantic salmon [42], since Ag^+^ enters into gill cells via copper transporters and sodium channels, while AgCl_0_(aq) may be taken up by simple diffusion [43].

In the case of AgNP exposure, the algal cells again had 2.5–4 times more cell-associated silver than the fish cells at 2.5–5 μM of external AgNP concentration (Fig. 1). We attribute this difference to a higher overall exposure of the algal cells. There might be various factors influencing the level of cell-associated silver, e.g. kinetics of internalization into fish cells, sorption differences, ongoing dissolution at the interface between AgNP and cell surface, and abundance of metal transporters. Indeed, algae cells were exposed in suspension, allowing AgNP and AgNO_3_ to interact from all sides with the cell surface (643 µm^2^/cell). In contrast, the fish cells were exposed as a cell monolayer sitting on a cell culture surface, which means only one side of the fish cells (half of the cell surface: 286 µm^2^/cell) was in immediate contact with AgNP or AgNO_3_.

### AgNP and silver ions elicit toxicity to algae and fish cells

**Fig. 2:**
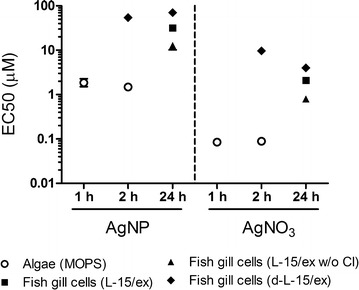
EC50 values of AgNP and AgNO_3_ in algae and fish cell exposures as a function of total silver. Times of exposure were selected based on the response of the respective cell type, with algal cells responding quickly with no further change in EC50 after 1 h whereas EC50 further decreased for fish cells over a 24 h period. Data presented as mean ± SD; n = 3. The *error bars* are smaller than the symbols due to the exponential scale in Y-axis

The photosynthetic yield was assessed to study the time-dependent toxicity of AgNP and AgNO_3_ in algae. The photosynthetic yield is an important parameter for evaluating the viability of algal cells as autotrophic organisms. In the fish cells, the overall metabolic activity was used as an endpoint upon AgNP and AgNO_3_ exposure. Effective concentrations causing a 50% decline (EC50s) in photosynthetic yield and metabolic activity were calculated from dose–response curves derived with algal and fish cells. The EC50s ranged from 1.5 to 1.9 µM (0.16–0.21 mg/L) AgNP in algal cells and from 12.7 to 70.3 µM (1.37–7.59 mg/L) AgNP in fish cells (Fig. 2). In AgNO_3_ exposures, EC50s were 0.09 µM (0.01 mg/L) in algae and 0.8–9.7 µM (0.09–1.05 mg/L) in the fish cells (Fig. 2). In the algae cell model, the EC50 values of AgNP determined in our study were comparable with EC50 values reported for other algal species [3, 44]. In the fish cell model, the EC50 values were similar to the EC50 values measured in other fish cell types [45, 46]. According to the categorization of toxic or non-toxic concentrations to aquatic organisms (<0.1 mg/L = extremely toxic; 0.1–1 mg/L = very toxic; 1–10 mg/L = toxic; 10–100 mg/L = harmful; <100 mg/L = non-toxic [47, 48]), we conclude that AgNP and AgNO_3_ are toxic to both algae and fish cells.

When comparing these EC50s, both similarities and differences were found for algal and fish cells. In terms of similarities, AgNP induced significantly lower toxicity than AgNO_3_ in both cell types if EC50s are expressed as a function of total silver present (Fig. 2). In terms of differences, algae were 10 to 100 times more sensitive than fish cells to both AgNP and AgNO_3_ exposure. On the other hand, if EC50s are expressed as a function of dissolved silver in the respective exposure media, it was found that the toxicity of AgNP to algae is mediated solely by dissolved silver [26] while in fish cells, AgNP were found to induce toxicity by a nanoparticle specific effect as well as dissolved silver [27].

The media composition also had a strong effect on the toxicity of AgNP to fish cells (Fig. 2) [27]. Among the three fish cell exposure media, AgNP yielded highest toxicity in L-15/ex w/o Cl and lowest toxicity in d-L-15/ex. This difference correlates with the degree of AgNP agglomeration in the media: the strongly agglomerated AgNP (size: 1000–1750 nm) in L-15/ex w/o Cl induced a 2-times higher toxicity than moderately agglomerated AgNP (size: 200–500 nm) in L-15/ex and 5-times higher toxicity than weakly agglomerated AgNP (size: 40–100 nm) in d-L-15/ex medium. Likely, the different degrees of agglomeration resulted in differing degrees of deposition of AgNP onto the fish cells, with the strongest agglomeration leading to highest cell exposure. This trend indicated that agglomeration and deposition could increase the interaction of AgNP with RTgill-W1 cell and induce higher toxicity. In the present exposure model, fish cells formed a cell layer on the bottom of the wells and AgNP suspensions were added on top. Previous modeling work showed that large size nanoparticles are transported faster than small nanoparticles due to deposition [49, 50]. Because of particle deposition on the cell monolayer, AgNP agglomeration may increase the interaction of AgNP with cells and thereby AgNP toxicity. Among the fish cell exposure media, d-L-15/ex medium maintains AgNP stability and better reflects the freshwater environment to which gill cells of freshwater fish would be exposed. Therefore, d-L-15/ex was selected to study the interaction of AgNP with fish cells in more detail.

**Fig. 3:**
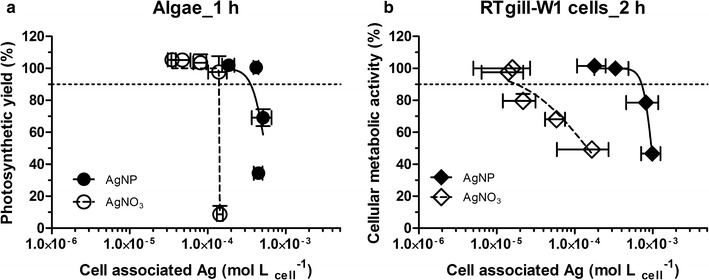
Recalculation of the toxicity of AgNP and AgNO_3_ to algae in MOPS **a** and fish cells in d-L15/ex medium **b** as a function of cell-associated silver. The *dashed horizontal lines* show the EC10 level. Each data point presents the mean of three independent experiments with the *horizontal lines* indicating the variation in cell-associated silver and the *vertical lines* the variation in effect (mean ± SD, n = 3). All data are expressed as % of the respective unexposed control. Data presented as mean ± SD; n = 3

The effects of AgNP and AgNO_3_ to algae and fish cells were recalculated as a function of cell-associated silver (Fig. 3; Additional file 1: Table S1). The EC10s (concentrations leading to 10% effect compared to unexposed control) were used for comparison because effects were quantifiable based on experimental data under all conditions for this level of effect (see horizontal dashed line in Fig. 3). The EC10 of AgNP and AgNO_3_ in algae are 1.40 × 10^−4^ and 3.55 × 10^−4^ mol/L_cell_, respectively. The EC10 of AgNP and AgNO_3_ in fish cells are 1.80 × 10^−5^ and 7.22 × 10^−4^ mol/L_cell_, respectively. In both cell models, the AgNO_3_ concentration response curve is left of the AgNP concentration response curve, indicating stronger effects of AgNO_3_. This can be interpreted as AgNP inducing toxicity via different mechanisms compared to AgNO_3_. However, this difference between AgNO_3_ and AgNP concentration response curves is much greater in the fish cells, demonstrating that fish cells respond strongly to a particle-specific impact, whereas in algae, dissolved silver is the dominant cause of toxicity.

The fact that no particle-specific effect was seen in algae suggested that AgNP were not incorporated into algae but that they may adhere to the algal surface from which Ag^+^ may dissolve and as such be taken up in the cells. To follow up on this hypothesis, the localization of cell-associated silver in algae was investigated by time-of-flight secondary ion mass spectrometry (TOF–SIMS), a qualitative and quantitative surface analysis. Indeed, the silver intensity from algae exposed to AgNP and AgNO_3_ indicated a strong sorption of AgNP onto the algal surface [26]. In contrast, fish gill cells take up AgNP in an energy—dependent process: as demonstrated by TEM coupled with energy-dispersive X-ray analysis that localized the NPs in the endocytic compartments of the cells [28]. This latter finding corresponds to the work of others who confirmed metal nanoparticles uptake by vertebrate cells via endocytic pathways [51–53].

### AgNP can bind cellular proteins and inhibit enzyme activity

Considering the contrasting finding that algal cells do not take up AgNP while fish cells do, and the importance of the AgNP-protein binding in nano-bio interaction, we postulate that extracellular proteins are more important in terms of AgNP exposure for algae while intracellular proteins should be considered for fish cells. One important extracellular protein is alkaline phosphatase, an enzyme responsible for phosphorus acquisition. This enzyme is highly abundant in aquatic environments, and is produced by a wide range of organisms including bacteria, fungi, zooplankton and algae [54–56]. Previously, alkaline phosphatase activity in periphyton was shown to be unaffected [57] or stimulated [58] by AgNP. However, considering that algae within periphyton are embedded in a matrix of biomolecules forming a biofilm [59], other factors might influence direct interaction of AgNP with the enzyme, e.g. periphyton community continuously synthesizes and secrets enzymes, it could not be determined whether the absence of inhibitory effects was due to a lack of interaction with AgNP or whether any impact was masked by de-novo synthesis of the enzyme.

Indeed, studying the interaction of AgNP or AgNO_3_ with isolated alkaline phosphatase showed that AgNP have a particle—specific, inhibitory effect on alkaline phosphatase activity and that this inhibition depends on the sequences of addition of enzyme substrate or AgNP [29]. Other studies have reported on an inhibitory effect on extracellular enzymes by nanoparticles. For example, in the same study on periphyton cited above [57], inhibition of β-glucosidase and l-leucine aminopeptidase was attributed to dissolved silver and particle specific effects. Inhibitory effects of the same proteins were also seen in heterotrophic biofilm exposed to titanium dioxide nanoparticles [60]. Thus, understanding the mechanisms of interaction of extracellular proteins and nanoparticles in general is an important future direction.

In order to study the interaction of AgNP with intracellular proteins in fish cells, the AgNP-protein corona was recovered from intact endocytic compartments by a newly established method with subcellular fractionation. Proteins acquired from the AgNP-protein corona were identified by mass spectrometry and analyzed with Gene Ontology. A total of 383 proteins were identified in this way and broadly classified as belonging to cell membrane functions, uptake and vesicle-mediated transport and stress-response pathways [28]. These proteins regulate substance transport across the plasma membrane or play key roles in cell metabolic processes, such as Na^+^/K^+^-ATPase, Ca^2+^-ATPase, adaptor-related protein complex 1 (AP-1B1), caveolin 1, flotillin 1/2, EH-domain containing protein 1/2/4 and Rab Family Small GTPases (RAB5A, RAB7A, RAB18) [28]. Based on the identified proteins, the processing of AgNP in fish cells was reconstructed: AgNP were taken up by fish cells via endocytic processes and stored in endosomal/lysosomal compartments [28]. Binding to AgNP could impair the function of these proteins and subsequently disrupt the normal cell activity, which would relate to the decline of cell viability in RTgill-W1 cells exposed to AgNP. Some of these proteins were also identified in the corona of magnetic nanoparticles exposed to human lung epithelial cells (A549) and HeLa cells [24, 25]. This indicated that vertebrate cells take up metal nanoparticles via common pathways regardless of elemental composition or coating of particles, exposure conditions and cell types.

**Fig. 4:**
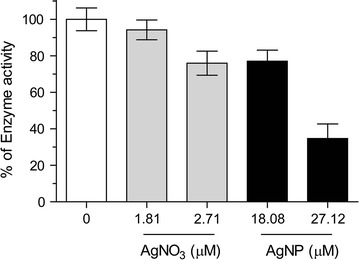
Inhibition of Na^+^/K^+^-ATPase activity by AgNO_3_ and AgNP. The concentration of Na^+^/K^+^-ATPase was 0.5 U/mL (19.5 µg/mL) in all experiments; the concentration of AgNO_3_ was selected based on the concentration of dissolved silver in AgNP suspension. Both a silver ion as well as a particle-specific effect was found with the latter being more dominant. Figure was reproduced from Yue et al. [28] with permission from the Royal Society of Chemistry. Data presented as mean ± SD; n = 3

Among the proteins identified from fish cells, Na^+^/K^+^-ATPase was selected to study the effect of AgNP on corona proteins. Experiments on the isolated, single protein showed that the inhibition of enzyme activity is attributable primarily to a particle-specific rather than a dissolved silver ion effect (Fig. 4). Schultz reported that citrate coated AgNP, i.e. the same type of particle used in this work, led to a particle-specific inhibition of Na^+^/K^+^ ATPase activity in juvenile rainbow trout gill in vivo [61]. Thus, our in vitro study was confirmative of the findings in vivo and signifies a strategy to further investigate AgNP and other nanoparticles for their interaction with corona proteins, based on the protein list established as described above.

## Conclusion

The results of the side by side comparison of AgNP-cell interactions for algal and fish cells are summarized in Table 2. The composition of exposure media influenced AgNP behavior and toxicity, highlighting once more the importance of characterizing nanoparticle speciation in the risk assessment of nanomaterials. Because of the barrier surrounding the cell membrane, AgNP cannot be taken up by algal cells but adsorb onto the cell surface instead, and toxicity is thus induced by dissolved silver. On the other hand, in fish cells, AgNP are taken up via endocytic pathways, causing toxicity by both dissolved silver and a nanoparticle specific effect. Thus, the cell type and structure are important features to be considered in nanotoxicity research. AgNP can bind extracellular and intracellular proteins and inhibit enzyme activities via nanoparticle specific effects. Current work provides a first concrete attempt to study the interaction of AgNP with extracellular and intracellular proteins from aquatic organisms. For future assessment, this kind of knowledge not only aids in mechanism-based aquatic risk assessment but also helps designing safer nanoparticles.

**Table 2:** Summary of the interaction of AgNP with algae and fish cells

	Algae	Fish cell
NP behavior	Slight agglomeration in MOPS medium	Slight agglomeration in d-L-15/ex medium and strong agglomeration in L-15/ex and L-15/ex w/o Cl media
Toxicity	Dissolved silver^a^	Dissolved silver^a^, nanoparticle-specific effect
Cellular uptake	No, adsorbed on algal surface	Yes, uptake via endocytic pathways
AgNP-protein interaction	Adsorption of extracellular enzyme on AgNP, inhibition of enzymatic activity	Cell membrane proteins and endocytic proteins binding to NP, inhibition of enzymatic activity
Cell structure and AgNP association		

^a^Dissolved silver indicates all the silver species in oxidized state Ag(I) in aqueous solution, such as Ag^+^, AgCl_n_ (aq) and AgOH (aq), stemming from AgNP

## Methods

### AgNP preparation and characterization

The citrate coated AgNP were purchased from NanoSys GmbH (Wolfhalden, Switzerland) as aqueous suspension with a concentration of 1 g/L (9.27 mM referring to the total silver, pH 6.46). The Z-average size and Zeta potential of AgNP in the stock solution were 19.4 nm and −30 mV, respectively. The stock AgNP solution was kept in the dark. A stock solution (10 mM) of AgNO_3_ (Sigma-Aldrich, Switzerland) was prepared by dissolving AgNO_3_ in nanopure water (16–18 MΩ/cm; Barnstead Nanopure Skan AG, Switzerland). The experimental solutions of AgNP and AgNO_3_ were freshly prepared by adding AgNP and AgNO_3_ stock solution into the respective exposure media and vortexing for 10 s. Unless specifically indicated, all chemicals were purchased from Sigma-Aldrich. As the work focused on the impacts of AgNP on algae and fish cells, we chose the respective AgNP and AgNO_3_ concentrations to observe significant effects in the toxicity experiments. In the uptake experiments, the exposure concentrations were selected to meet the ICP-MS detection limit.

The AgNP were characterized in nanopure water and under experimental conditions in each exposure media. The Z-average size and zeta potential of the AgNP were measured by dynamic light scattering (DLS) and electrophoretic mobility using a Zetasizer (Nano ZS, Malvern Instruments, UK) [26, 27]. To measure the dissolution of AgNP in exposure medium, dissolved silver was separated by two methods: centrifugal ultrafiltration with a nominal molecular weight cut-off of 3 kDa (Amicon ultra-4 centrifugal filter units, Millipore, Germany) centrifuged at 3000×*g* for 0.5 h (Megafuge 1.0R, Heraeus Instruments, Germany) and by ultra-centrifugation (CENTRIKON T-2000, KONTRON Instruments, Switzerland) at 145,000×*g* for 3 h. The silver concentration was measured by Inductively Coupled Plasma Mass Spectrometry (ICP-MS, Element 2, Thermo Finnigan, Germany). The dissolution of AgNP was calculated by dividing the measured dissolved silver concentration to the related nominal total silver concentration. The average of the AgNP dissolution (Table 1) obtained by ultra-filtration and ultra-centrifugation was used for recalculation of the concentration–response curves as a function of dissolved silver (Fig. 3).

### Algal culture and exposure of cells

The alga *E. gracilis* strain Z (Culture Collection of Algae, Göttingen, Germany) was cultured in the Talaquil medium (pH 7.5) supplemented with vitamins B1 and B12 [62] at 20 °C under light–dark cycles of 12 h each on a shaker (Infors, Switzerland) with 90 rpm. The cell number and volume were measured by a particle counter (Beckman Coulter Z2, USA).

Before exposure to AgNO_3_ and AgNP, exponentially grown algae were centrifuged at 2000×*g* for 10 min and then re-suspended in MOPS. The final cell density for toxicity assessment was 1.5 × 10^4^ cell/mL. After exposure to AgNO_3_ (0–400 nM) and AgNP (0–40 μM) for 1 and 2 h, to study toxic effects, the photosynthetic yield was measured by fluorometry using a PHYTO-PAM (Heinz Walz GmbH, Germany) [26]. The values were presented as percentage of controls, and were plotted as a function of measured total silver and cell-associated silver [26].

### RTgill-W1 cell culture and exposure of cells

RTgill-W1 cells were routinely cultivated in L-15 medium (Invitrogen, Switzerland), supplemented with 5% fetal bovine serum (FBS, Gold, PAA Laboratories GmbH, Austria) and 1% penicillin/streptomycin (Sigma-Aldrich, Switzerland) in 75 cm^2^ flasks. The L-15 medium containing these supplements is termed “complete L-15”. Cells are routinely cultured in the dark in normal atmosphere at 19 °C.

For exposure to AgNO_3_ (0–5 μM) and AgNP (0–100 μM), cells were seeded in 24-well microtiter plates or 25 cm^2^ flasks, and cultured in complete L-15 medium. After being fully confluent, cell monolayers were washed with either L-15/ex, L-15/ex w/o Cl or d-L-15/ex. Then, 1 mL/well of AgNP or AgNO_3_ suspension in the respective media was added to culture wells. Exposure proceeded for 2–24 h at 19 °C. AlamarBlue (AB, Invitrogen, Switzerland) was used to measure the cellular metabolic activity to assess the toxicity of AgNP to fish gill cells [27, 32]. Before incubation with AlamarBlue, the AgNP suspension was removed and exposed cells were carefully washed with PBS to removed loosely adsorbed AgNP. Control experiments showed no interference of the silver with the AlamarBlue assay.

### Uptake of AgNP by algae and fish cells

The algae were exposed to AgNP (0–10 μM) and AgNO_3_ (0–500 nM) at a cell density of 1 × 10^5^ cell/mL in order to meet the detection limit of the ICP-MS. After 1 h of exposure, the algae were washed to remove loosely bound AgNP with fresh medium or adsorbed silver ions by cysteine using the following protocol: algae exposed to AgNP or AgNO_3_ were first centrifuged (2000×*g*, 10 min) and resuspended in MOPS. After 2 wash cycles, the algae were re-suspended in cysteine–MOPS and gently stirred for 5 min. After washing, the algae were filtered (SM 16510, Sartorius) and digested for metal analysis [26].

To quantify the fish cell-associated silver, RTgill-W1 cells were cultured in 25 cm^2^ flasks until confluency and then exposed to AgNP (2.5–10 μM) or AgNO_3_ (0.5–5 μM) in d-L-15/ex medium. After exposure, the medium with AgNP or AgNO_3_ was removed and cells were washed twice with cysteine for 5 min. Cells were then trypsinized. Detached cells were re-suspended in complete L-15 medium. Cell suspensions were centrifuged at 1000×*g* for 3 min to pellet the cells. Cell pellets were re-suspended in 550 µL PBS and the cell density determined by an electronic cell counter (CASY1 TCC, Schärfe System, Germany). A volume of 500 µL cell supernatant was digested for metal analysis [28].

Samples from algae and fish cell exposures were digested with 4.5 mL of 65% HNO_3_ in a high-performance microwave digestion unit (MLS-1200 MEGA, Switzerland) at a maximum temperature of 195 °C for 20 min. The digests were diluted 50-times and measured by ICP-MS. The detection limit for ICP-MS quantitation of silver was 10 ng/L (1.0 × 10^−5^ mol/L_cell_ in the current work). The reliability of the measurements was determined using specific water references (M105A, IFA-Tull, Austria). As the volume of algal cells is larger than that of the fish cells, the measured cell associated silver was related to cell volume and expressed as mol/L_cell_ in order to be able to directly compare the cell-associated silver in algae and fish cells. The associated silver was also related to the cell number and expressed as mol per cell to be able to compare with other reports.

The localization of cell-associated silver in algae was checked by time-of-flight secondary ion mass spectrometry analysis (TOF–SIMS, ToF.SIMS 5 instrument, ION-TOF GmbH). The primary ion was 25 keV Bi^+^ to ensure high sensitivity to silver and the sputtering time was 5.2 s leading to ablation of a few nanometers of the surface layer of the cell [26]. The AgNP uptake in fish cells was investigated by transmission electron microscopy (TEM, FEI Morgagni 268, 100 kV) and energy dispersive X-ray (EDX) spectroscopy analyses in a scanning transmission electron microscope (STEM, Hitachi HD-2700) [28].

### Interaction of AgNP with proteins

Alkaline phosphatase (Sigma-Aldrich) was select as a representative extracellular algal enzyme to study the interaction with AgNP. The effect of AgNP to alkaline phosphatase was assayed in MOPS by determining enzyme activity, using fluorescently linked 4-methylumbelliferyl phosphate disodium salt as substrate [29].

In fish cell exposures, to identify the proteins binding to AgNP in cells, AgNP-protein corona complexes were recovered from intact subcellular compartments isolated by subcellular fractionation and proteins lysed from the AgNP to be detected by mass spectrometry. The identified proteins were analyzed by DAVID (http://david.abcc.ncifcrf.gov/), a protein ontology analysis tool. Among the identified proteins, Na^+^/K^+^-ATPase (Sigma-Aldrich, No. A7510) was selected to study the interaction of AgNP with intracellular proteins. The effect of AgNP on Na^+^/K^+^-ATPase activity was measured in a buffer containing 20 mM Tris–HCl, 0.60 mM EDTA, 5 mM MgCl_2_, 3 mM KCl and 133 mM NaCl (pH 7.8) and substrate, ATP (Sigma-Aldrich, No. A9062) [28].

### Data analysis

All data were analyzed by GraphPad Prism (version 5.02 for Windows, USA). Fluorescent units obtained in the cell assays were converted to percent viability of control cells. Concentrations leading to 10% and 50% effect (EC10s, EC50s) were determined by nonlinear regression sigmoidal dose–response curve fitting using the Hill slope equation, and were presented as the mean of three independent experiments, with a 95% confidence interval. Data presented as mean ± standard deviation, n = 3.

## Additional file

**Additional file 1: Table S1.** EC10 and EC50 values, corresponding 95% Confidence Intervals, Hill slope and R^2^ of AgNP and AgNO_3_ concentration-response curves.
