# Supplementary material for: Interaction of silver nanoparticles with algae and fish cells: a side by side comparison
Source: J Nanobiotechnology. 2017 Feb 28;15:16. doi: 10.1186/s12951-017-0254-9 (PMC5331694; doi:10.1186/s12951-017-0254-9)
Supplement: Supplementary file 1 — Additional file 1: Table S1. EC10 and EC50 values, corresponding 95% Confidence Intervals, Hill slope and R2 of AgNP and AgNO3 concentration-response curves. [file 12951_2017_254_MOESM1_ESM.docx]

**Supplementary Information**

**Interaction of silver nanoparticles with algae and fish cells: a side by side comparison**

Yang Yue^1,2,¶,*^, Xiaomei Li^1,2^, Laura Sigg^1,3,#^, Marc J-F Suter^1,3^, Smitha Pillai^1,3^, Renata Behra^1,3,*^, Kristin Schirmer^1,2,3,*^

^1^ Eawag, Swiss Federal Institute of Aquatic Science and Technology, Department of Environmental Toxicology, Dübendorf, CH 8600, Switzerland.

^2^ École Polytechnique Fédérale de Lausanne, School of Architecture, Civil and Environmental Engineering, Lausanne, CH 1015, Switzerland.

^3^ ETH-Zürich, Department of Environmental Systems Science (D-USYS), Zürich, CH 8092, Switzerland.

^¶^Current address: Department of Basic Sciences and Aquatic Medicine, Norwegian University of Life Sciences (NMBU), Oslo, O 0454, Norway

^#^Current address: Wattstrasse 13a, 8307 Effretikon, Switzerland

*Corresponding authors: Yang Yue, email: [yang.yue@eawag.ch](mailto:yang.yue@eawag.ch); Renata Behra, email: [Renata.Behra@eawag.ch](mailto:Renata.Behra@eawag.ch); Kristin Schirmer, email: [kristin.schirmer@eawag.ch](mailto:kristin.schirmer@eawag.ch)

**This supplementary material contains the following sections:**

Table S1: EC10 and EC50 values, corresponding 95% Confidence Intervals, Hill slope and R^2^ of AgNP and AgNO_3_ concentration-response curves.

Table S1: EC10 and EC50 values, corresponding 95% Confidence Intervals, Hill slope and R^2^ of AgNP and AgNO_3_ concentration-response curves.

|  |  | EC10 (mol L_cell_^-1^) | EC50 (mol L_cell_^-1^) | 95% confidence interval of EC50 | Hill Slope | R^2^ |
| --- | --- | --- | --- | --- | --- | --- |
| Alage photosynthetic yield | AgNO_3_ | 1.40E-04 | 1.42E-04 | 1.40E-04 to 1.43E-04 | -170.8 | 0.9799 |
|  | AgNP | 3.55E-04 | 5.49E-04 | 3.51E-04 to 8.58E-04 | -5.043 | 0.3434 |
| Fish gill cell metabolic activity | AgNO_3_ | 1.80E-05 | 1.46E-04 | 1.08E-04 to 1.98E-04 | -1.049 | 0.8731 |
|  | AgNP | 7.22E-04 | 9.65E-04 | 9.52E-04 to 9.79E-04 | -7.565 | 0.9917 |
